# Visualizing healthcare system variability and resilience: a longitudinal study of patient movements following discharge from a Swedish psychiatric clinic

**DOI:** 10.1186/s12913-020-05642-3

**Published:** 2020-08-25

**Authors:** Jakob Svensson, Johan Bergström

**Affiliations:** Division of Risk Management and Societal Safety, Lund University, Box 118, 221 00 Lund, Sweden

**Keywords:** Patient safety, Healthcare, Psychiatry, Discharge, Resilience, Performance variability, Time-lapse, Visualization

## Abstract

**Background:**

As healthcare becomes increasingly complex, new methods are needed to identify weaknesses in the system that could lead to increased risk. Traditionally, the focus for patient safety is to study incident reports and adverse events, but that starting point has been contested with a new era of safety investigations: the analysis of everyday clinical work, and the resilient healthcare.

This study introduces a new approach of system monitoring as a way to strengthen patient safety and has focused on discharge in psychiatry as a risk for adverse outcomes. The aim was to analyse a psychiatric clinic’s everyday ‘normal’ performance variability of discharge from inpatient psychiatric care to outpatient care.

**Method:**

A retrospective longitudinal correlation study with a strategic selection. Data consist of 70,797 patient visits within one psychiatric clinic, and the visits were compared between 81 different wards in Stockholm County by using a model of time-lapse visualization.

**Results:**

The time-lapse visualization shows a discrepancy in types of visits and the proportion of cancelled visits to the outward units. 42% of all patients that were scheduled as an outward patient, did not complete this transition, but instead, they revisit the clinics’ emergency ward and did not receive the planned care treatment. The patients who visit the emergency ward instead of their planned outpatient visit did this within 20 days.

**Conclusions:**

The findings show a potential increased demand for emergency psychiatric care from 2010 to 2018 within the clinic. It also suggests that the healthcare system creates a space of temporal as well as functional variability, and that patients use this space to adapt to their changing conditions. This understanding can assist management in prioritising allocation of resources and thereby strengthen patient safety. Today’s incident reporting systems in healthcare are ineffective in monitoring patterns of more cancelled visits in outward units and sooner visit to the emergency ward. By using time-lapse visualization of patient interactions, stakeholders might analyse current-, and estimate future, stressors within the system to identify and understand potential system migration towards risk in healthcare. This could help healthcare management understand where resources should be prioritized.

## Background

In the late 1990s, patient safety evolved into discipline of its own in healthcare [1]. Today, patient safety is defined by the World Health Organization (WHO) as; “the absence of preventable harm to a patient during the process of health care and reduction of risk of unnecessary harm associated with health care to an acceptable minimum” [2]. Similar to WHO’s perspective that health ‘is more than the absence of disease’, patient safety ‘is more than the absence of accidents’ [3]. When an accident occurs, investigators in health care analyse what went wrong and how recurrence can be avoided. Traditionally, the focus for patient safety is to study the unexpected, the rare events that attract our attention [3], but that starting point of investigating adverse events and incident reports has been contested with a new era of safety investigations: the analysis of everyday clinical work and the resilient healthcare [4, 5].

In safety research, resilience is often defined as a capacity to adapt to emerging risks in complex environments [6]; to understand resilience is to understand performance variability and pressure [5, 7]. In this context, *performance variability* ‘is based on the principle of equivalence of “successes” and “failures” and the principle of approximate adjustments’ [8]. This means that there are degrees of freedom, also referred to as a ‘capacity of manoeuvre’ [9], in the strategies used to achieve acceptable outcomes. In this paper, we present a method for capturing patterns of organisational performance variability. The method visualizes a system’s (a psychiatric clinic – Stockholm Centre of Dependency Disorder) way of absorbing and adapting to pressure; i.e. its resilience. To do this, we focused the study on the organisational level of everyday clinical work of patient discharge from inpatient care to outpatient care. Classic safety management strategies are based on the idea of increased efficiency, i.e. work can be reduced to specific predefined tasks (routines and guidelines), hereby minimizing unwanted deviations and as a consequence, increase safety. However, contemporary safety research has moved away from this way of thinking and acknowledges the complexity of work [3]. Instead of trying to predefine workmanship, by limiting the degrees of freedom of work, safety can be strengthened by enhancing people’s (individually and collectively) capacities to adapt in uncertain circumstances [7]. This is the analytical starting point in contemporary studies of system resilience [10].

In Swedish healthcare, incident reporting is a common starting point for patient safety strategies since it is mandatory under current legislation. Yet, that only captures a small fraction of occurred events [11], and there is little evidence that incident reports enable organisational change [12]. A large number of incident reports are often interpreted as something positive, but the amount of data can also foster an illusion of a healthy patient safety culture as the organisation has numbers to lean against [13]. Incident reporting is not likely a productive way to understand everyday organisational performance and visualize where capacity stressors occur. Often the response to an adverse event in healthcare is to try to standardize processes, eliminate contributory factors and improve barriers [14]; i.e. limit performance variability. Although incident reports might be used as a search for cause and for improvement in the quality of care to insure that similar incidents can be avoided in the future. This paper provides another technique to visualize organisational risk without using incident reports.

**Fig. 1:**
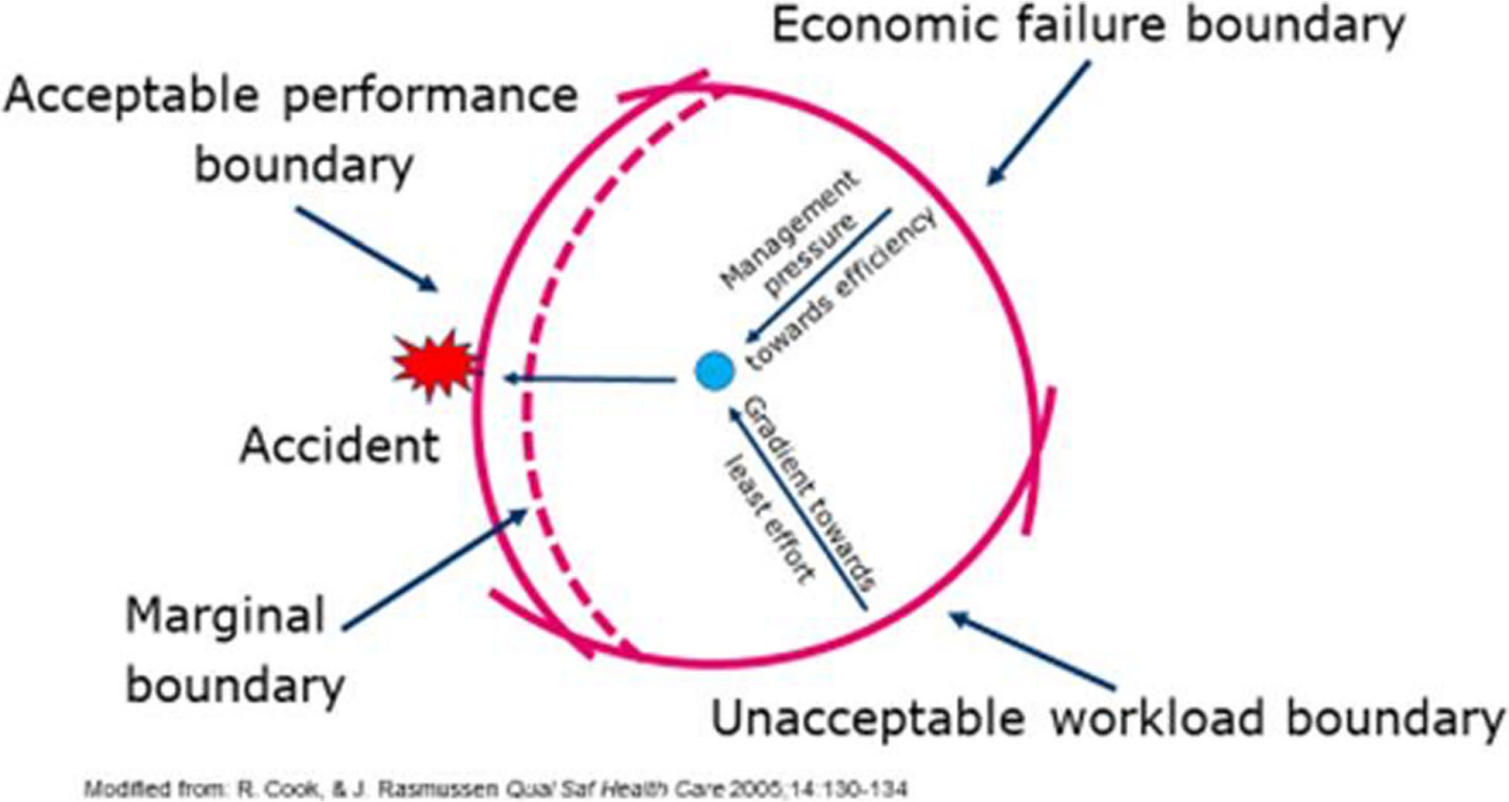
Dynamic safety model. Modified from Cook R, & Rasmussen J. Qual Saf Health Care 2005;14 [2]:130–134. It addresses how current conditions in healthcare lead to accidents over time. Gradients push the operation point towards the acceptable performance boundary and increase the risk of crossing the boundary (failure)

In the early 1980s, Rasmussen, a professor of system safety and human factors, took a new, naturalistic and complexity-based approach to safety when he theorised safety as emerging from the dynamic interactions of everyday performance [15]. He distanced himself from the behaviouristic concept of humans as predictable by stimuli-response. Instead, he described humans as goal-driven, selecting their goals, adapting to the environment and adjusting their actions according to what goes right [15]. In a naturalistic approach, the work environment should be designed in a way that helps people cope with the unexpected [16]. Accident investigations could thereby shift the focus from locating accountability and drawing moral boundaries to analysing why it made sense for people to do what they did at a particular time, and what trade-offs benefited their goal. Consequently, patient safety investigations should take the healthcare staff’s point of view, focusing on the disorganised details of everyday work. Rasmussen uses a dynamic safety model [ [16], Fig. 1] to introduce how economic pressure, (i.e., management’s pressure for increased efficiency) alongside striving to minimize workload, push the system and the everyday clinical work, towards the error margin and the boundary for acceptable performance.

The model in this study was built on Rasmussen’s theory of pressure over time and introduces and visualizes the performance variability of normal work. Such performance variability includes, how systems continuously adapt their operational point to meet the sometimes multiple and conflicting goals of operation, and what degrees of freedom, below referred to as ‘capacity of manoeuvre’ [9], (in Rasmussen’s model represented by the operational space constrained by the three boundaries), they have to make such adjustments. The theoretical heritage of this view comes from cybernetics and Ashby’s law of requisite variety [17]; essentially stating that in order to control a system the number of states in its control mechanisms must be at least equal to the number of potential system states. Safety scientists are interested in how such capacities of manoeuvre are achieved often without the system providing the necessary requisite variety in the first place, and in the (fundamentally human) “requisite imagination” [18] which implies the creative imagination of potential future system states in times of great adversity and stress. However, in modern healthcare, the pressure for increased efficiency has resulted in more control systems and less flexibility in daily work [19]. The idea that this would lead to a safe system is based on the impression that safety is achieved by constraining performance variability by limiting the degrees of freedom, which is typically done through the proceduralisation of work [13, 20]. Such assumptions are questioned in the wider literature [5, 10] on the resilience of complex high-risk systems, such as healthcare. This literature sees performance variability as the source of both safety and risk, as something that needs to be controlled rather than constrained [21].

This study focused on system resilience connected to a specific risk in healthcare, when patients are discharged from psychiatric inpatient care and are expected to continue treatment as an outpatient. In the literature, discharge in psychiatry is identified as a risk for suicide or adverse outcomes [22, 23]. It is also highlighted by the studied clinic’s (Stockholm Centre of Dependency Disorder) action plan for increased patient safety, as well as in surveys^1^ conducted by Stockholm Health Care Services, where the staff was asked what they consider to be the most significant patient safety risk in psychiatry.

In resilient healthcare research, a micro-level approach and qualitative research design are frequently used [6]. However, to study resilience as a complex adaptive system, Berg, Akerjordet, Ekstedt and Aase [24] suggested research on the meso- and macro-levels. Furthermore, from a complexity perspective, considering dimensions of time and space becomes highly important for understanding patterns of work [25]. Studying resilience at a meso-level through new methodological tools can benefit the understanding of organisational strain [26]. Consequently, this paper visualizes retrospective discharge and compares findings of variability within the Stockholm Centre of Dependency Disorder’s different wards.

## Method

The analysis was made possible by using a time-lapse model of patient visits. Registrations of patient visits in outward units were captured over 9 years and shown rapidly in a series so that slow action (such as system drifts) appears to happen quickly. The stakeholders can thereby, in a matter of seconds, get an estimate of performance variability (within a well-functioning clinic) across all outward units. The model facilitates the monitoring of ordinary meso-level patient discharge through a new system feedback tool. The ability to anticipate organisational pressure and allocate resources could thereby be strengthened. This is the first study to use time-lapse visualization for discharge data in psychiatry. Time-lapse has previously been used in e.g. medicine and biology to show dynamic anatomical or cellular development but has not been used in psychiatry for organisational purposes. By using time-lapse visualization, this study targets a new approach to looking at patient safety over time.

### Aim

The aim of this study was to analyse a psychiatric clinics everyday ‘normal’ performance variability of discharge from inpatient psychiatric care to outpatient care. Patterns were analysed from one clinic in Stockholm County where all patients had been discharged from inpatient care and scheduled as outpatients between 1 January 2010 and 31 December 2018.

### Context

The Stockholm Centre for Dependency Disorders is part of specialized psychiatry in Stockholm County. The clinic is organized within Stockholm County Councils and provides outpatient and inpatient healthcare, as well as emergency care for patients with addiction or substance dependency. This clinic is the largest psychiatric clinic in Stockholm and the main patient groups are people with addiction to alcohol, illegal drugs or pharmaceuticals. The clinic had 81 different units across the county during the studied time period. The lead author is employed within the studied clinic, which favoured our ability to extract relevant data.

### Description of research design

The study was conducted as a retrospective longitudinal correlation study with strategic selection. New sources of information were needed to support management in healthcare and to understand performance variability at the meso-level.

The data consist of 19,857 anonymized patients who had been hospitalized within the studied clinic as inpatients and who, at the time of discharge, had received an outpatient follow-up visit within the clinic. The analysis involves visualizing the day-to-day discharge process and its variability. The focus for the developed method was to support the quality improvement of system performance measurement.

### Data extraction

**Fig. 2:**
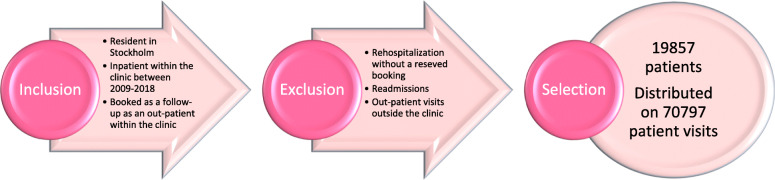
The selection process with inclusion and exclusion criteria

To understand the clinic’s *performance variability*, the study used quantitative data to visualize patterns over time. Data was obtained by designing a data extraction code based on the inclusion and exclusion criteria (Fig. 2). The code was developed in the autumn of 2018 by analysing incident reports and the result from Stockholm County Council’s surveys of patient safety culture. A master copy of the data was transferred to Excel, and the data selection contained 19,857 patients distributed on 70,797 patient visits. Every patient had been discharged from inpatient care and scheduled as an outpatient. Only those who were residents in Stockholm and only reservations within the Stockholm Centre of Dependency disorders were included. Since the aim was to visualize retrospective discharge in the clinic, discharges that were followed by readmissions without a reservation to an outward unit were excluded. Visualizing a specific selection of patients enabled a comparison over time of how the clinic has adapted to potential fluctuations of patients visits to outward units. Therefore, the discharges that did not contain a subsequent reservation to outpatient care were excluded.

Microsoft SQL Server Management Studio was used for the extraction of the data.

### Data analysis method

**Fig. 3:**
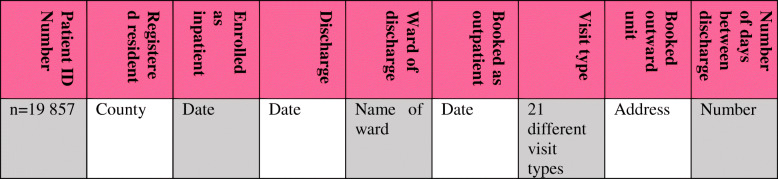
The categories for the data assessment table, which consisted of 70,797 lines in Excel

The analysis aimed to illustrate system variability and to synthesize tendencies. In the first phase, every patient was given a unique number starting with 1, 2, 3 …, etc. up to a total of 19,857. By listing the patient numbers, the authors could identify how many times a specific patient (number) had been scheduled as an outpatient. Each number was listed in individual lines with data under each category (Fig. 3). The name of the ward was used as a control instrument for the data. If an abnormality arose, it could be determined from which ward the discharge took place in order to further investigate the deviance. No deviances occurred from the data sample, and later on, the result did not take into account which ward was responsible for the discharge.

In the second phase, visualizations were conducted through Microsoft 3D Maps for Excel. This programme is a three-dimensional data visualization tool where geographic and temporal data can be shown over time. Data was entered into a worksheet and the columns were represented as layer panes. When adding an exact location in the worksheet, the programme showed the added data as staked columns in accurate position on a map. By using this instrument, the authors could speed-up patient visit, intercept, zoom-in and out on different wards and analyse patterns in different locations and phases within the studied timeframe. Tendencies were disclosed in each outward unit and visualization of the clinics every day clinical work emerged. Through the visualization, five characteristics of outward visits appeared as particularly interesting and brought out further investigation (Table 1). The authors went back to the raw data and separated the variables that emerged through the visualization as unexpected or that needed clarification to present the fluctuations by each year. These characteristics were linked to the selection of patients and are, in this study, an example of how time-lapse visualization can display variability over time. The data in Table 1 only presents the amount for each year, whereas visualization can provide a deeper understanding by showing every patient visit quickly over time.

**Table 1:** Five different variables that emerged from the visualization as specific notable aspects from the discharge from inpatient care to outpatient care. Characteristics of outward visits within the clinic

Variables	Year								
	2010	2011	2012	2013	2014	2015	2016	2017	2018
**Number of outward reservations from inpatient care to outpatient care**	7680	8340	8066	7844	7666	8020	8034	7738	7409
**Days between inpatient care and the reserved outward visit (median)**	9	6	5	6	5	5	7	7	5
**Did not show up/late cancellation to the reserved outward visit**	119	177	259	327	366	436	509	484	530
**Median number of days until next emergency ward visit**	27	25	30	31	26	20	18	17	10
**Visits to the emergency ward instead of the reserved outward visit (number)**	2988	3331	2945	3086	3126	3509	3769	3692	3389
**Visits to the emergency ward instead of the reserved outward visit (%)**	39.91%	39.94%	36.51%	39.34%	40.78%	43.75%	46.91%	47.71%	45.74%

The study does have some limitations. It did not evaluate the quality of care, nor assess the outcome of a received treatment. The visualization did not reveal effects of what time the outward visit was planned, why some ward units had more cancelled visits than others, or why some units had fewer visits in total. Contextual factors, such as patient status or individual ward units’ specific commissions, were not taken into account. The purpose of the visualization was to get a deeper understanding of inconstancies in the follow-up care and create a starting point for patient safety measurement and evaluation of system pressure.

## Results

**Fig. 4:**
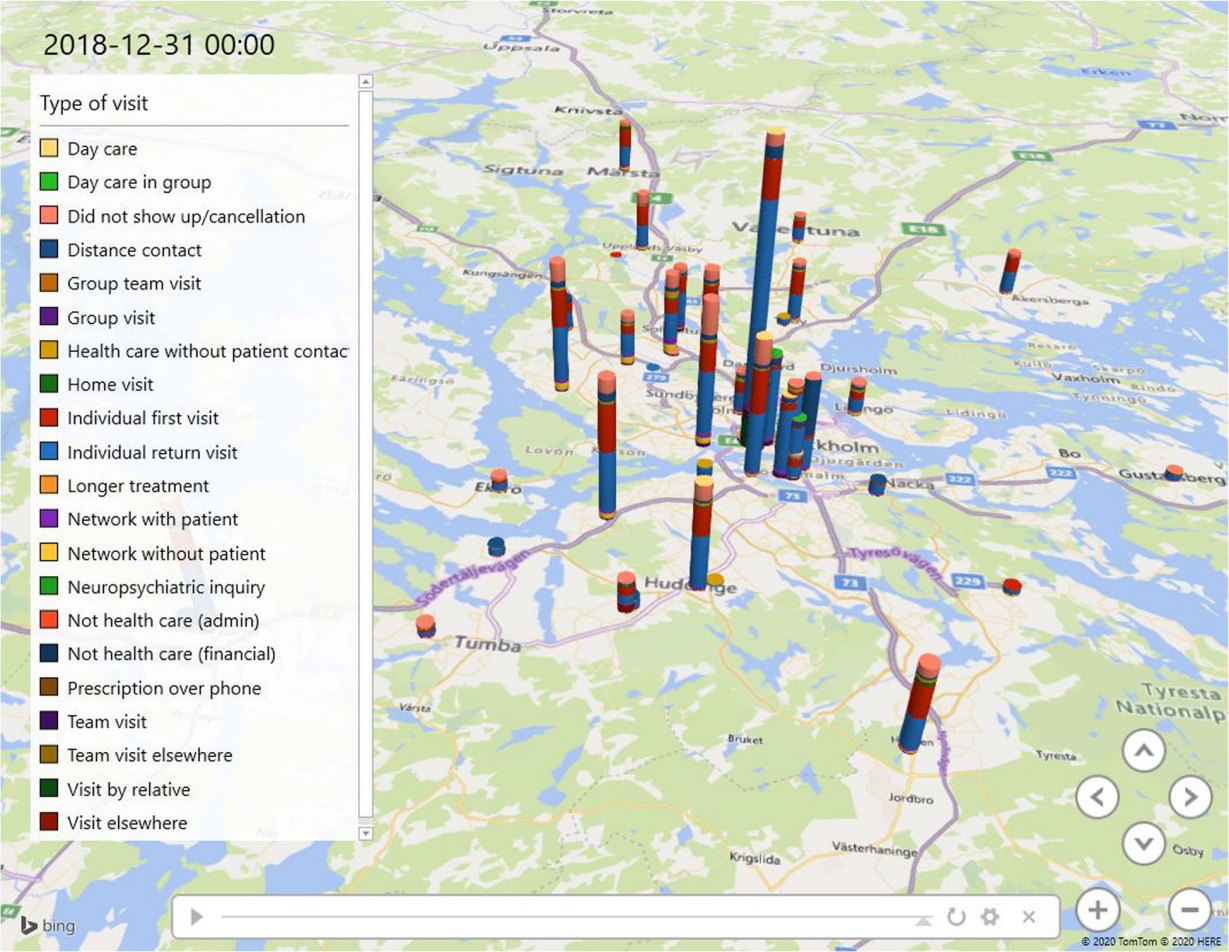
Patient visits accumulated under 10 years. Visits to the emergency ward in the clinic have been excluded in this image (Microsoft product screen shot reprinted with permission from Microsoft Corporation)

**Fig. 5:**
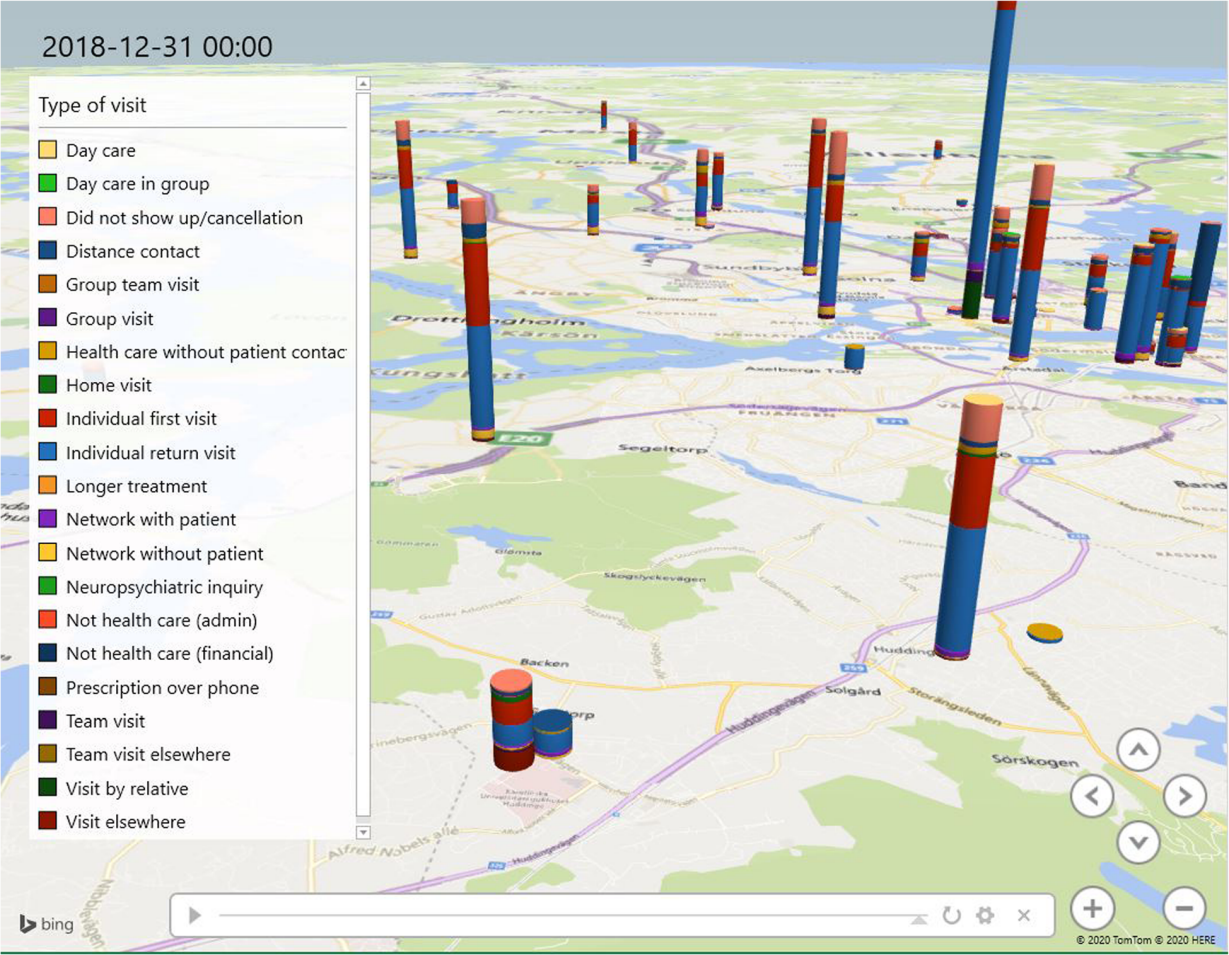
Zoom-in on specific wards in a specific time to contrast variability (Microsoft product screen shot reprinted with permission from Microsoft Corporation)

**Fig. 6:**
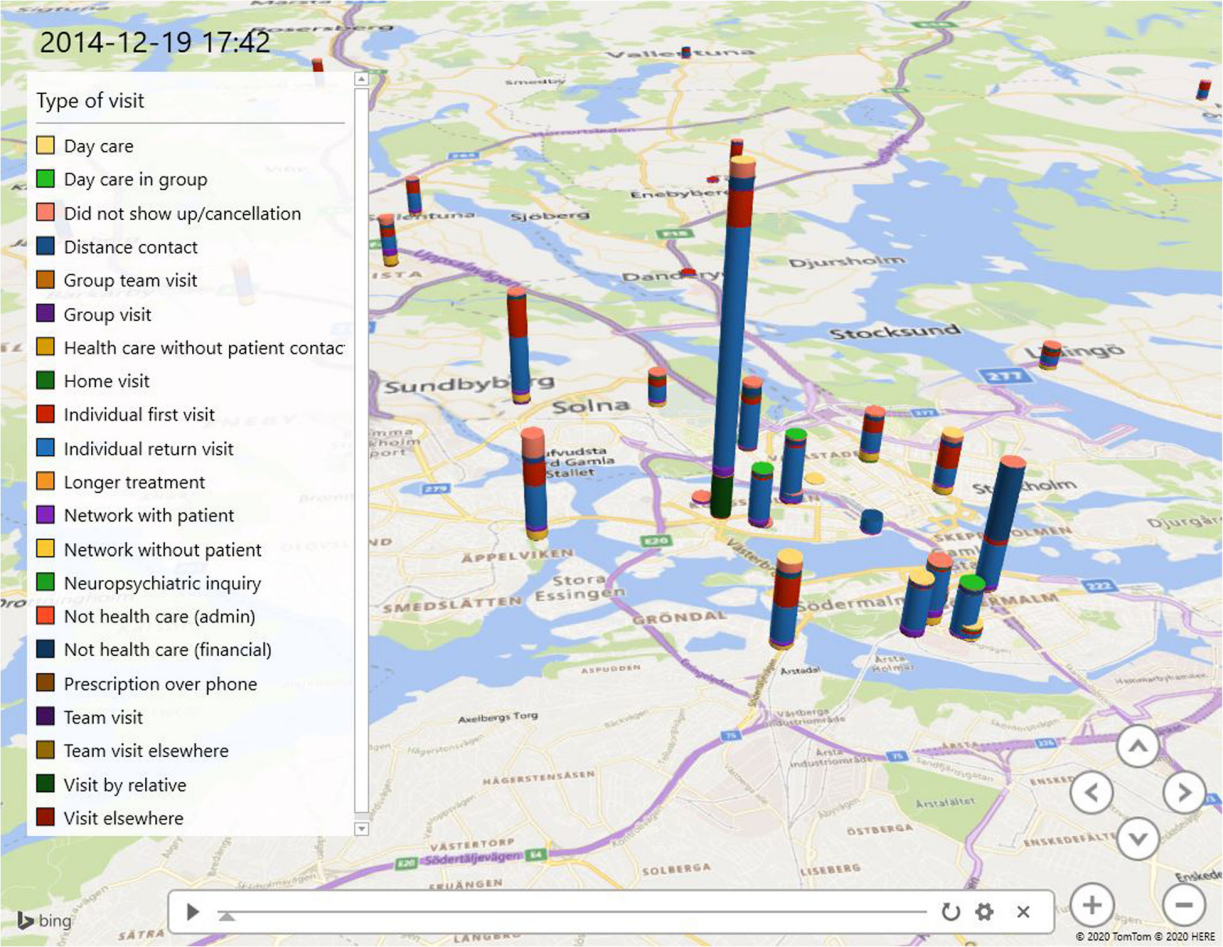
Shows a specific date (2014-12-19). The visualization can target a certain time and place, which can be used by the clinic to follow up if specific changes where implemented and if these changes had an effect on the studied ward units (Microsoft product screen shot reprinted with permission from Microsoft Corporation)

The visualization shows the patients’ paths through psychiatric healthcare. Figures 4, 5 and 6 illustrate outward units within the Stockholm Centre for Dependency Disorder distributed across Stockholm County. The figures are only snapshots and, in the visualization, the bars (which represent the location of the outward units) grow over time, and it is possible to zoom-in and pinpoint each individual ward unit and find out statistics on different patient visits. The different colours represent different types of visits. The model illustrates how normal healthcare outward transfer occurs.

Figures 4, 5 and 6 show examples of three different situations where the visualization tool can present an easy comparison of the studied selection. This study used discharge from inpatient care, but the visualization can be used in many other circumstances to discover variability and to study ‘normal work’.

At first glance, in Fig. 4, the wards seem to have a similar distribution of visit-types across the county. However, the visualization shows a discrepancy in most of the visit types. Cancelled visits fluctuate in different outward units, whereas the availability for specific healthcare most likely affects the patient’s motivation to show up. Some units, for example, are specialized in drug-assisted treatment, which favours compliance and thereby has a smaller number of cancelled visits.

A particularly interesting pattern that became detectible in the visualization was when a certain patient visited the emergency ward instead of the scheduled outward visit; i.e. when the patient had a reserved time for the outward visit, but the next visit was instead to the emergency ward. This pattern was seen in 42% of the scheduled outward visits from all 70,797 patient visits. The clinic’s ambition is to offer a visit to the outward unit the following day. The visualization did not reveal whether the emergency ward visits were affected by when the outward visit was planned. However, it was most common that the patient visited the outward unit the following day. By having a close timeframe for the outward visit, patients can start their treatment sooner and reduce their length of illness. The idea is that this would enhance the likelihood of a patient showing up and receiving adequate treatment. Different substance use disorders require different treatments; nevertheless, the common denominator for the studied patients was that all were expected to visit the outward unit, and not the emergency ward, for their next healthcare visit.

The visualization showed changes in patient visits that occurred within the clinic. As seen in Table 1, the number of outward reservations from inpatient care to outpatient care fluctuated over the 9 years. From 2010 through 2018, the number of cancelled outward visits increased each year; at the same time, the median number of days until the next emergency ward visit decreased. The number of data points in Table 1 is not intended for a distinction between common cause versus special cause variation. This study used visualization as a tool for understanding unanticipated risk areas within a healthcare system. The numbers in Table 1 are variables that the visualization illustrates through the time-lapse technique.

## Discussion

The visualization shows that a large portion (42%, from 2010 through 2018) of all patients scheduled as outward patients, does not complete this transition; instead they revisit the emergency ward and do not receive the planned treatment (at least not at the planned time and place). The patients who visited the emergency ward instead of their planned outpatient visit, did this, on average, within 20 days (calculated from median value). This tells us that there is both temporal (in terms of when a patient is scheduled for an outward visit as well as when the patient actually seeks care) and functional (in terms of what kind of care the patient seeks, the emergency ward or the outward unit) performance variability in the process of revisiting the clinic. The system seems to allow for such performance variability by offering a capacity of manoeuvre [9], manifested by the degrees of freedom and possibilities to make visits to the emergency ward rather than to receive the planned treatment. Additionally, as our data suggests, the patients use this capacity to adapt to their changing conditions. This means that the clinic offers an opportunity for the patients to shift from their planned treatment, based on their condition.

Schubert, Wears, Holden and Hunte [27] discussed the concept of patients as experts in detecting problems in their condition and suggested that their knowledge should be seen as a source for enhancing system resilience. The variability in the patient visits illustrates the interplay between the micro- (patient) and meso- (clinic) levels of the system. Further, the analysis indicates a patient demand that can be anticipated through the model and stakeholders/managers could use compensatory strategies [28] to provide appropriate resources to meet this performance variability.

This study did not analyse the quality of care, nor possible causes for the data in Table 1. To state a clear cause would require more in-depth analysis of e.g. the reasons for the emergency visit rather than attending the scheduled outpatient visit. The study instead introduces an approach to analysing organisational adaptive capacities [19], that is, sources and patterns of resilience. It is not intended to be a normative judgement regarding whether or not such resilience is desirable. As emphasised in the background section, according to resilience engineering theory, performance variability is the source of both success and failure and should be controlled rather than constrained [21]. Visualizations of the kind this study introduced could lead to a discussion about the implications of everyday clinical work and how, given the patterns of variability, to prioritize and allocate resources.

According to The Swedish National Board of Health and Welfare’s management system for systematic quality work [29], incident reports should be accumulated to enable the healthcare provider to see patterns that indicate weaknesses in healthcare quality. As discussed, the incident reporting system does not allow for the identification of patterns that reflect everyday performance. The model presented in this paper is based on statistics from authentic patient visits and interactions and could therefore uncover a more dependable starting point for patient safety analysis.

This study focused on system resilience and the risk in patient safety when patients are discharged from psychiatric inpatient care with continued treatment as outpatients. The findings indicate a potential increased demand for emergency psychiatric care from 2010 through 2018. The combination of earlier visits to the emergency ward, more cancelled visits in outward units and an increase in revisits to the emergency ward after discharge, create new stressors for the system. Today’s reporting systems in healthcare are ineffective in monitoring such patterns. The use of time-lapse visualization could simplify the analysis of system drifts towards acceptable performance boundaries. Questions that arise from the time-lapse visualization include whether the emergency ward has adapted accordingly, for example, with increased staffing or number of patient beds, or whether the increase has merely increased workload and demand for adaptation at the level of emergency ward staff. Working conditions evolve over time, and by monitoring the system at the meso-level, migrations by the system [30], as described in Rasmussen’s Dynamic Safety Model [16], could be identified. The potential drifts towards more cancelled visits in outward units and earlier visits to the emergency ward, migrate the clinics operating point. By monitoring interactions over time instead of reacting to incident reports, larger system changes could be identified, and management can evaluate whether adaption regarding resources has changed accordingly.

The ability to anticipate challenges and be proactive could be characterized as an expression of resilience [4, 31]. To do this might foster a different patient safety system. The care provider has a legal obligation to develop methods for follow-up and analysis of quality and safety within the management system [29]. Chapter 3 in the Swedish Patient Safety Act [32] states that the healthcare provider must ‘take the necessary measures to prevent patients from adverse events’ and ‘decide on measures to prevent similar incidents from occurring again’. Requirements like this are typically met by an increased bureaucratization of patient safety with additional levels of regulation, control systems and routines [13], that is, a reduction in performance variability.

A further focus on understanding, anticipating and providing capacity of manoeuvre [9] could also prevent the ward from ‘going solid’ [19], i.e. tightly coupled condition with no buffer for an increase of patient visits. By using time-lapse visualization of normal performance variability, the clinic could possibly foresee the outcome of remission, and thereby, plan ahead for when patients will return to the emergency ward. This could in prolongation enhance the patient’s ability to regain health during hospitalization and further encourage a discussion of shared decision-making [33]. The results highlight questions of whether the idea of scheduled visits to the outward units is the right way to organize care for the patient with dependency disorder, at least within the timeframe in which such visits are currently planned. More personal continuity in outward units could possibly affect the frequency of outward visits [34].

The model visualizes separate ward units and their proportion of patient visits. Indeed, there is a risk that it might be used normatively to identify ‘underperforming’ units within the clinic. However, the data does not suggest why some ward units have more cancelled visits than others or why some units have fewer visits in total. The result does not contain contextual factors, such as patient status or individual units’ specific commissions. Instead, the study provides a methodology to generate meso-level patterns from micro-level interactions. Organisations should study these interactions as a way to examine system resilience and patient safety.

## Conclusions

As healthcare becomes increasingly complex, new methods are needed to identify weaknesses in the system that could lead to an increased risk in patient safety. This study introduces a starting point of system monitoring as a way to strengthen patient safety. Retrospective analysis of a large amount of micro-level interactions seems to be a valuable tool for widening the understanding of everyday clinical work and risk. In this study, 70,797 patient visits by 19,857 patients were aggregated in a visualization showing the patterns by which patients interact with psychiatric care following discharge. The findings suggest that the healthcare system creates a space of temporal as well as functional variability and that patients use this space to adapt to their changing conditions. This understanding can assist management in prioritising the allocation of resources and thereby strengthen patient safety. By visualizing patient interactions within a clinic, stakeholders might analyse current stressors and estimate future stressors within the system to identify and understand potential system migration towards risk in healthcare. The study used a time-lapse visualization of everyday ‘normal’ clinical work, rather than incident reports or root-cause analysis, as a method to understand patterns of resilience and how risk could emerge in a psychiatric clinic. The findings in this study indicate that risk emerges over time in everyday normal organisational performance. To confirm these findings future research should include a micro-level approach, for example, the patients’ views on why the scheduled outward visits differ from where they are actually seeking care.

## Data Availability

The datasets are available from the corresponding author on reasonable request.

## Abbreviations

WHO: World Health Organization

## Footnotes

1 Internal survey carried out by the county council. All employees (*n* = 700) within the clinic were given the opportunity to answer a questionnaire with several claims about perceived patient safety.
